# Kidney-Targeted Epoxyeicosatrienoic Acid Analog, EET-F01, Reduces Inflammation, Oxidative Stress, and Cisplatin-Induced Nephrotoxicity

**DOI:** 10.3390/ijms22062793

**Published:** 2021-03-10

**Authors:** John D. Imig, Md Abdul Hye Khan, Anna Burkhan, Guan Chen, Adeniyi Michael Adebesin, John R. Falck

**Affiliations:** 1 Drug Discovery Center and Cardiovascular Center, 8701 Watertown Plank Road, Milwaukee, WI 53226, USA; astavniichuk@mcw.edu; 2 Department of Pharmacology & Toxicology, Medical College of Wisconsin, Milwaukee, WI 53226, USA; gchen@mcw.edu; 3 Department of Biochemistry, University of Texas Southwestern Medical Center, Dallas, TX 75390, USA; adeniyi.adebesin@utsouthwestern.edu (A.M.A.); j.falck@utsouthwestern.edu (J.R.F.)

**Keywords:** kidney-targeted, chemotherapy, nephrotoxicity, novel therapy, epoxyeicosatrienoic acid

## Abstract

Although epoxyeicosatrienoic acid (EET) analogs have performed well in several acute and chronic kidney disease models, targeted delivery of EET analogs to the kidney can be reasonably expected to reduce the level of drug needed to achieve a therapeutic effect and obviate possible side effects. For EET analog kidney-targeted delivery, we conjugated a stable EET analog to folic acid via a PEG-diamine linker. Next, we compared the kidney targeted EET analog, EET-F01, to a well-studied EET analog, EET-A. EET-A or EET-F01 was infused i.v. and plasma and kidney tissue collected. EET-A was detected in the plasma but was undetectable in the kidney. On the other hand, EET-F01 was detected in the plasma and kidney. Experiments were conducted to compare the efficacy of EET-F01 and EET-A for decreasing cisplatin nephrotoxicity. Cisplatin was administered to WKY rats treated with vehicle, EET-A (10 mg/kg i.p.) or EET-F01 (20 mg/kg or 2 mg/kg i.p.). Cisplatin increased kidney injury markers, viz., blood urea nitrogen (BUN), N-acetyl-β-(D)-glucosaminidase (NAG), kidney injury molecule-1 (KIM-1), and thiobarbituric acid reactive substances (TBARS). EET-F01 was as effective as EET-A in decreasing BUN, NAG, KIM-1, TBARS, and renal histological injury caused by cisplatin. Despite its almost 2×-greater molecular weight compared with EET-A, EET-F01 was comparably effective in decreasing renal injury at a 10-fold *w*/*w* lower dose. EET-F01 decreased cisplatin nephrotoxicity by reducing oxidative stress and inflammation. These data demonstrate that EET-F01 targets the kidney, allows for a lower effective dose, and combats cisplatin nephrotoxicity. In conclusion, we have developed a kidney targeted EET analog, EET-F01, that demonstrates excellent potential as a therapeutic for kidney diseases.

## 1. Introduction

Renal disease in cancer patients and survivors has emerged as a major healthcare issue and continues to escalate sharply [1,2,3]. Newer cancer therapies have increased cure rates and survival times, but therapy-associated fluid and electrolyte abnormalities and acute kidney injury progressing to chronic kidney disease are common sequelae [3,4,5]. Anti-cancer drugs, vascular endothelial growth factor receptor tyrosine kinase inhibitors (VEGFRI) and platinum derivatives can also cause severe renal injury that significantly compromises their effective and safe use [3,4,5]. There is a significant clinical need for novel therapies to effectively treat and stop kidney disease in cancer patients. This healthcare issue has led to the development, evolution, and emergence of a subspecialty, Onco-Nephrology [5]. Current treatments only slow the loss of kidney function, or have no benefit at all [2,5,6]. New approaches are urgently needed. Our ongoing research is attacking this emerging cancer arena and we are focused on developing epoxyeicosatrienoic acid (EET) analogs to protect the kidney from cancer therapy-associated toxicity. 

EET analogs are based on the pharmacophoric moiety of EETs and designed with improved solubility and resistance to auto-oxidation and metabolism by soluble epoxide hydrolase (sEH) [7,8]. Previous studies have demonstrated that synthetic EET analogs are kidney protective in several pathological conditions like hypertension, drug-induced nephrotoxicity, and lupus nephritis [9,10,11,12,13]. EET analogs decrease kidney damage in these disease states through vasodilator, diuretic, anti-inflammatory, anti-oxidative, and anti-apoptotic activities [10,11,12,13]. These studies clearly demonstrated that an EET-based approach combats kidney damage in pathological situations of different etiologies. Recently, attempts have been made to develop EET analogs that possess the unique property to target the kidney. A novel form of kidney targeted EET analog was designed and synthesized by conjugating the EET pharmacophore with folic acid (EET-F01). In the present study, we investigated the ability for EET-F01 to target the kidney when compared to the EET analog, EET-A. EET-F01 and EET-A were compared for their ability to decrease cisplatin-induced kidney injury. Lastly, this study further delineated the kidney protective anti-inflammatory and anti-oxidative actions of EET-F01. 

## 2. Results

### 2.1. EET-F01 Targets Kidney Tissue

**Figure 1:**
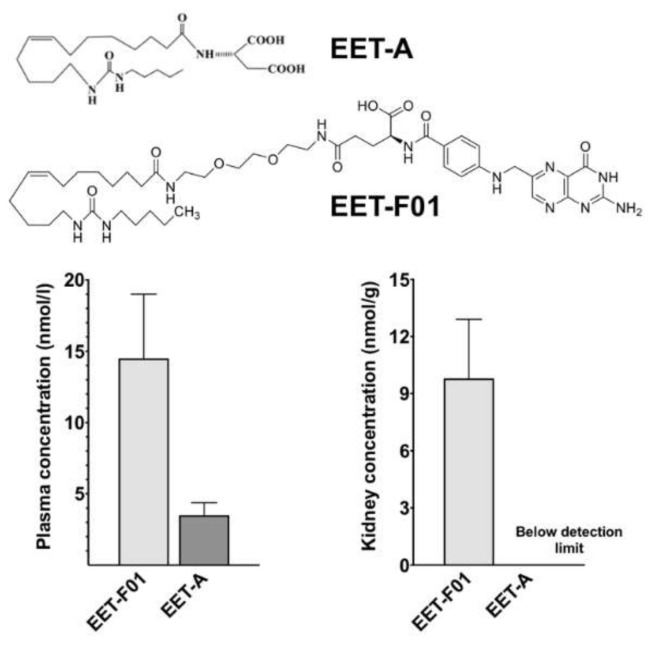
Comparison of plasma and kidney EET-F01 and EET-A following i.v. administration for 6 h. Top panel: Chemical structures for EET-A and EET-F01. Left panel: Plasma concentration of EET-F01 and EET-A. Right panel: Kidney levels of EET-F01 and EET-A. Data expressed as mean ± SEM, *n* = 3/group.

Chemical structures for EET-A and EET-F01 are provided in Figure 1. Purity for EET-A and EET-F01 was >95% as determined by NMR. In the acute animal study, equal doses by weight of EET-A and EET-F01 were administered intravenously for 6 h followed by plasma and kidney tissue collection. We demonstrated 5-fold higher plasma level of EET-F01 compared to EET-A after 6-h continuous administration (Figure 1). Interestingly, we also demonstrated that after 6-h continuous administration the kidney content of EET-A was almost un-detectable compared to EET-F01 content in the kidney (Figure 1). These data demonstrate that EET-F01 targets the kidney.

### 2.2. EET-F01 Protects against Cisplatin Nephrotoxicity

**Figure 2:**
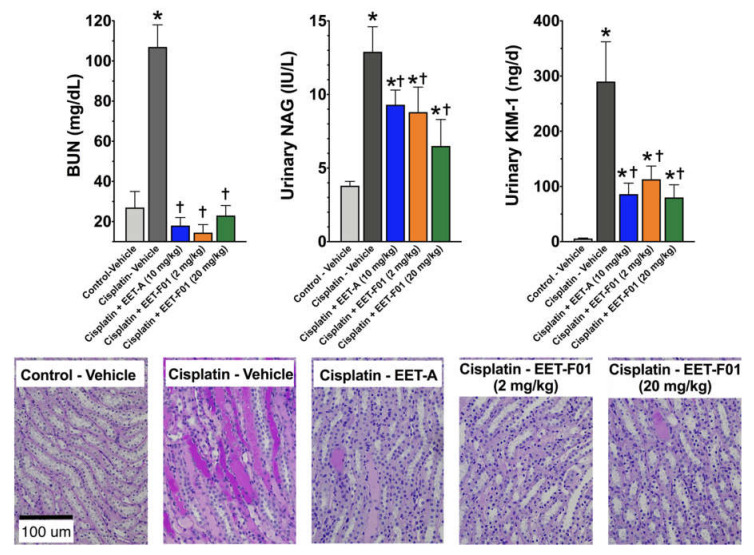
Kidney injury induced by cisplatin is reduced by EET-F01 and EET-A treatments. Left panel: blood urea nitrogen (BUN), Middle panel: urinary N-acetyl-β-(D)-glucosaminidase (NAG), Right panel: kidney injury molecule-1 (KIM-1) in cisplatin administered rats treated with vehicle, EET-F01, or EET-A. Bottom panel: Representative photomicrographs of Periodic acid-Schiff (PAS) Staining depicting vacuolation and desquamation of the renal epithelial cells along with severe intra-tubular proteinaceous cast tubular cast formation in the renal sections of different experimental groups. * *p* < 0.05 vs. control–vehicle group; † *p* < 0.05 vs. cisplatin–vehicle group. Data expressed as mean ± SEM, *n* = 6/group.

In a separate set of experiments, groups of rats were pre-treated with EET-F01 and EET-A for two days prior to induction of cisplatin nephropathy and then further treated with the test compounds for another 5 days. The kidney protective actions of EET-F01 and EET-A were determined from BUN levels, urinary NAG content, and urinary KIM-1 levels. EET-F01 was as effective as EET-A in decreasing BUN, NAG, KIM-1 and renal histological injury five days following induction of cisplatin nephrotoxicity (Figure 2). Next, histological examination was conducted to further assess cisplatin nephrotoxicity. Cisplatin administration resulted in tubular injury manifested by vacuolation and desquamation of the renal epithelial cells along with severe intra-tubular proteinaceous cast formation in both cortical and medullary regions of the kidney. EET-A and EET-F01 treatment protected the kidney from cisplatin nephrotoxicity in rats (Figure 2). EET-F01 was effective at 20 mg/kg/d and at a 10-fold lower dose of 2 mg/kg/d despite its almost 2×-greater molecular weight due to the folate compared with EET-A. These data clearly demonstrate that EET-F01 targets the kidney and facilitates a lower, yet still efficacious dose. Thus, we have developed a kidney targeted EET analog, EET-F01, that demonstrates excellent potential as a therapeutic for kidney diseases. 

### 2.3. EET-F01 and EET-A Reduce Renal Inflammation and Oxidative Stress in Cisplatin Nephrotoxicity

**Figure 3:**
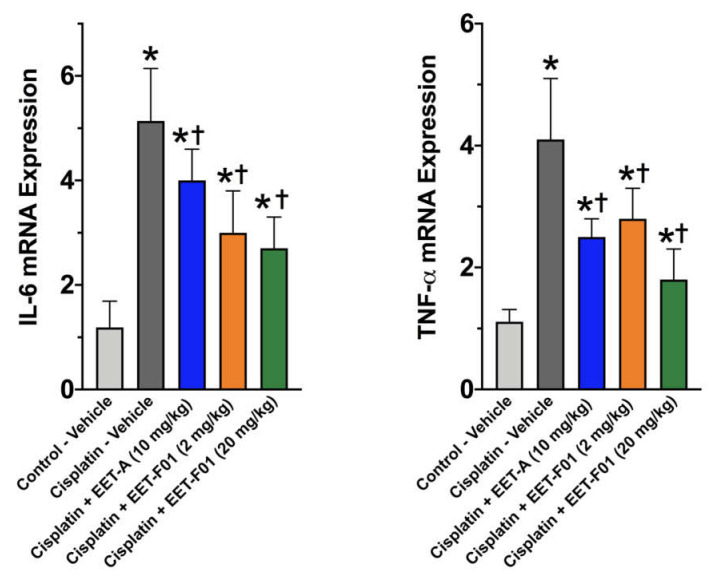
Renal inflammation in cisplatin nephrotoxicity is reduced by EET-F01 and EET-A treatments. RT-PCR analysis for mRNA expressions of IL-6 (left panel) and TNFα (right panel). * *p* < 0.05 vs. control–vehicle group; † *p* < 0.05 vs. cisplatin–vehicle group. Data expressed as mean ± SEM, *n* = 6/group.

**Figure 4:**
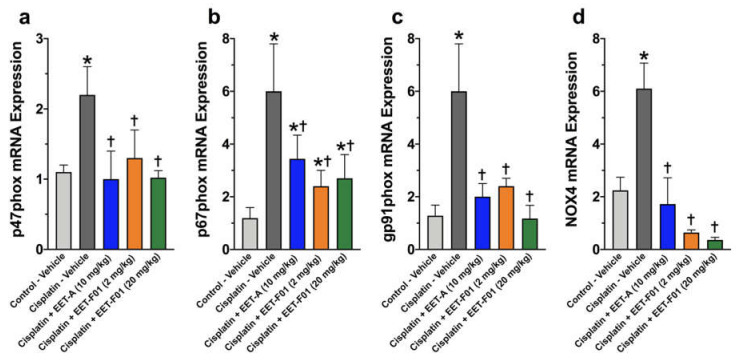
Renal oxidative stress in cisplatin nephrotoxicity is reduced by EET-F01 and EET-A treatments. RT-PCR analysis for mRNA expressions of (**a**) p47phox, (**b**) p67phox, (**c**) gp91phox, (**d**) NOX4. * *p* < 0.05 vs. control–vehicle group; † *p* < 0.05 vs. cisplatin–vehicle group. Data expressed as mean ± SEM, *n* = 6/group.

**Figure 5:**
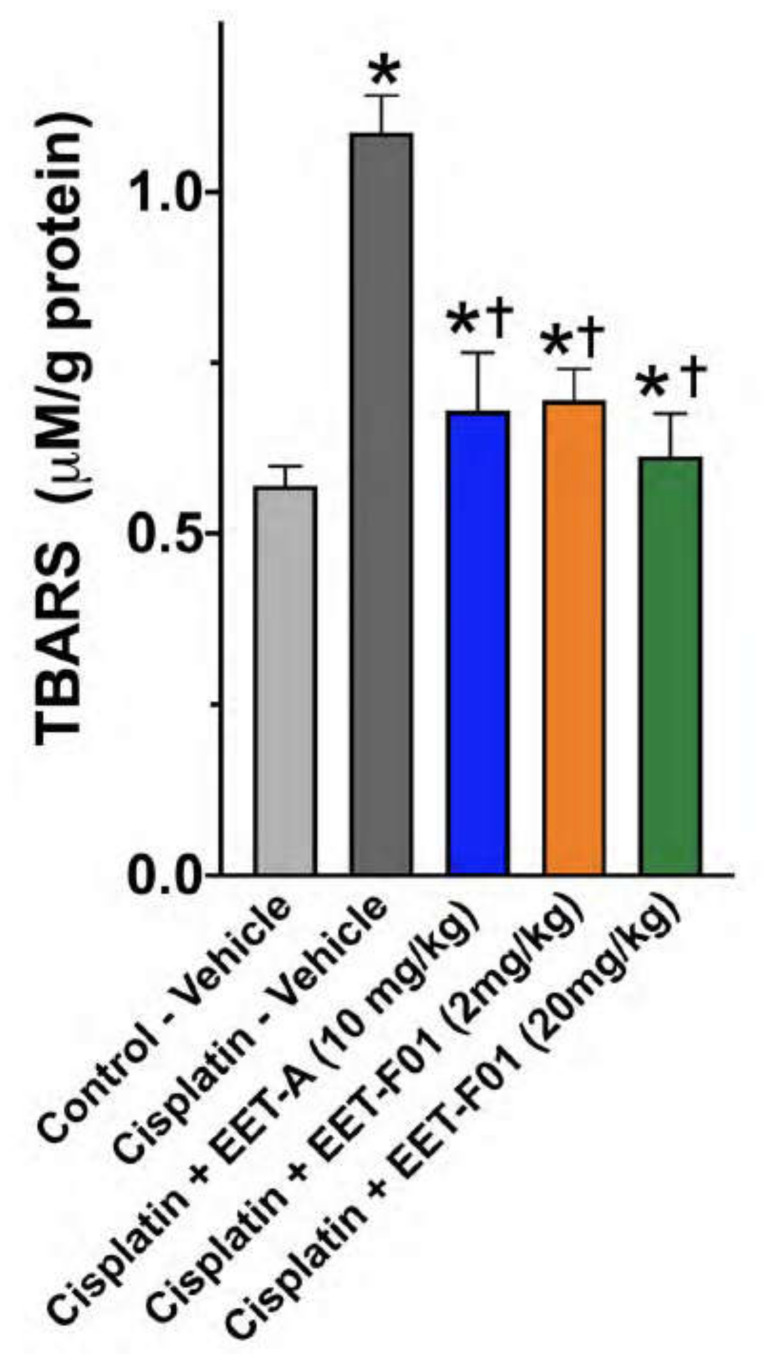
Kidney thiobarbituric acid reactive substances (TBARS) in cisplatin nephrotoxicity is reduced by EET-F01 and EET-A treatments. * *p* < 0.05 vs. control–vehicle group; † *p* < 0.05 vs. cisplatin–vehicle group. Data expressed as mean ± SEM, *n* = 6/group.

Renal IL-6 and TNFα mRNA expression levels revealed a 4 to 5-fold increase in cisplatin-induced nephrotoxicity. EET-F01 and EET-A treatment reduced renal inflammatory genes IL-6 and TNFα by 30–50% in cisplatin nephrotoxicity (Figure 3). Increased renal expression of the oxidative marker genes NADPH oxidase subunits p47phox, p67phox, gp91phox, and NOX4 is observed in cisplatin administered rats. Kidney tissue TBARS levels were increased during cisplatin-induced nephrotoxicity. These cisplatin-induced increases in oxidative stress were attenuated 60–100% by EET-F01 and EET-A treatment (Figure 4 and Figure 5). These data demonstrate that kidney targeted EET-F01 is as effective as EET-A in reducing inflammation and oxidative stress in cisplatin nephrotoxicity.

### 2.4. EET-A Does Not Enhance Tumor Growth

**Figure 6:**
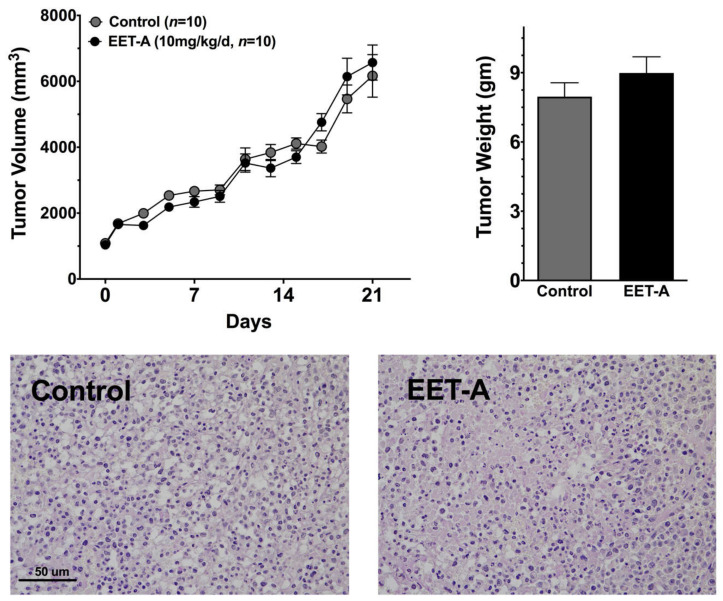
Tumor growth of a human breast cancer cell line, MBA-MB-231, in athymic mice is not altered by EET-A treatment. Left panel: tumor volume, Right panel: tumor weight in MBA-MB-231 tumor bearing mice treated with vehicle (control) or EET-A. Bottom panel: Representative photomicrographs of Periodic acid-Schiff (PAS) Staining depicting similar tumor hyperplasia and proliferation in human breast tumors. Data expressed as mean ± SEM, *n* = 5 mice and *n* = 10 tumors/group.

The use of EET analogs might promote cancer metastasis when administered for long periods of time [14,15]. Although the current therapeutic approach would administer EET analogs for a short period of time to prevent cisplatin-induced nephrotoxicity, we evaluated the long-term effects of EET-A on tumor growth of a human breast cancer cell line, MBA-MB-231, in athymic mice. Tumor volume and weight increased at the same rate in vehicle or EET-A treated mice over a three-week period (Figure 6). Histological evaluation of the tumors failed to demonstrate any changes in cellular hyperplasia or proliferation in EET-A treated mice. These data demonstrate that EET-A does not enhance tumor growth.

## 3. Discussion

Chemotherapeutic agents are extremely important in combating cancers; however, organ /toxicities remain a significant adverse side-effect [4,5,6]. Cisplatin is a widely prescribed chemotherapeutic agent for a variety of malignancies [1,2,16]. Nephrotoxicity that involves acute kidney injury that progresses to chronic kidney disease is the most common and severe organ toxicity associated with cisplatin therapy [4,5,6,17]. Cisplatin nephrotoxicity occurs because epithelial cells uptake cisplatin which results in renal inflammation, oxidative stress, and tubular damage [4,5,6,17]. Previous studies have demonstrated that EET analogs can attenuate renal damage [10,11]. In this study, we demonstrate that a kidney targeted EET analog, EET-F01, decreases cisplatin induced renal inflammation, oxidative stress, and tubular damage, but at a weighted and molar dose significantly lower than EET-A. 

Targeted delivery of drugs to the kidney has ample literature precedent and this strategy can be reasonably expected to reduce the level of drug needed to achieve a therapeutic effect in the target organ and ameliorate side effects, if any are detected [18,19]. For instance, selective, kidney-targeted delivery of drugs can be achieved by conjugation to folic acid, due to the high concentration of folate receptors in renal tissue [20,21]. The concept that folate-conjugation of pharmaceuticals can target the kidney has been demonstrated for rapamycin and tempol [18,19,22]. Folate is coupled to drugs to permit the cellular uptake of the drug by folate receptor mediated endocytosis [23,24] Unlike the folate receptor, the folate carrier that is widely expressed in tissues does not uptake folate conjugated drugs [24]. Fortunately, in the kidney the folate receptor-α (folbp1) is highly expressed in the proximal tubule [25]. Folate-conjugated drugs have been successful targeted to the kidney by taking advantage of this high level of folate receptor-α expression in the proximal tubule [18,19,22]. We anticipated that EET analogs would behave similarly to these other drugs when folate conjugated. 

Our scientific research group has previously demonstrated that EET analogs have great promise for treating acute and chronic kidney diseases [11,13,26,27]. EET analog actions include vasodilation, inhibition of platelet adhesion, blood pressure lowering action that takes days to weeks, and long-term anti-inflammatory, anti-oxidative, and anti-fibrotic actions over the course of weeks to months [26,27,28]; thus, EET analogs are poised to treat serious and life-threatening kidney diseases in patients. Kidney targeted EET analog drugs would also be advantageous because they would allow a reduction in the drug level needed to achieve efficacy, decrease side-effects, and prevent potential interference with systemic cisplatin actions. As proof-of-principle, an EET analog backbone connected to folic acid via an ethyleneoxydiamine linker, EET-F01, was synthesized and tested. EET-F01 demonstrated kidney targeting when compared to the EET analog, EET-A. 

Next, EET-F01 was compared to EET-A for attenuation of cisplatin nephrotoxicity. In the current study, we clearly demonstrate that EET-F01 decreases levels of renal injury markers BUN, NAG and KIM-1 in cisplatin nephrotoxicity. Kidney histology also revealed decreased tubular damage following cisplatin administration. More importantly, despite an almost 2x-greater molecular weight compared with EET-A, EET-F01 was as effective in decreasing cisplatin nephrotoxicity at a 10-fold lower weighted dose. These results provide strong evidence that the kidney targeted EET analog, EET-F01, can achieve a therapeutic kidney protective action at a reduced drug level that warrants further translational study as a therapy and/or prophylactic for drug-induced nephrotoxicity. 

One mechanism for kidney protective actions of EET analogs is through reduced inflammation. Indeed, EET-A has previously been demonstrated to decrease renal inflammatory cell infiltration and levels of monocyte chemoattractant protein-1 (MCP-1), TNF-α, IL-6, and IL-1β in acute and chronic kidney disease models [11,12,26]. EET analogs decrease inflammation in unilateral ureter obstruction, lupus nephritis, and diabetic nephropathy animal models [11,29,30]. These studies also provided evidence that EET analogs prevent epithelial to mesenchymal transition and fibrosis that is responsible for progression of acute kidney injury to chronic kidney disease [29,30]. Likewise, inflammation is a key contributor to the acute to chronic kidney disease pathological changes during cisplatin nephrotoxicity [26,31,32]. These studies found IL-6, TNF-α, and IL-10 to be involved in progression of cisplatin nephrotoxicity [31,32,33]. Findings also demonstrated that EET-A did not interfere with cisplatin-induced tumor cell death [26]. In the current study, cisplatin induces increased renal expression of inflammatory biomarkers KIM-1, TNF-α and IL-6. EET-A and EET-F01 significantly reduced renal inflammation in rats with cisplatin nephrotoxicity. EET-F01 at 2 mg/kg/d was able to achieve an anti-inflammatory level similar to EET-A at 10 mg/kg/d. This clearly demonstrates that EET-F01 at a reduced drug level had comparable anti-inflammatory actions to combat cisplatin nephrotoxicity. 

Oxidative stress is another contributing factor to renal injury following cisplatin chemotherapy [26,31,33,34]. Cisplatin nephrotoxicity is accompanied by increased NADPH oxidase (NOX) activity [33,34]. These previous studies have demonstrated increases in renal NOX4, p47phox, and gp91phox in cisplatin nephrotoxicity [33,34,35,36,37,38]. We have previously demonstrated that EET analogs can effectively decrease oxidative stress [10,13,26]. and that EET-A decreased oxidative stress in cisplatin nephrotoxicity by reducing kidney MDA and gp91phox levels [20]. In the present study, we evaluated the ability for the kidney targeted EET analog, EET-F01, to decrease renal oxidative stress in cisplatin nephrotoxicity. We demonstrated markedly increased expression of the oxidative stress genes p47phox, p67phox, gp91phox and NOX4 in cisplatin nephrotoxicity. Interestingly, our study demonstrated that EET analogs, EET-F01 and EET-A, markedly reduced renal oxidative stress expression to a similar level. This is in agreement with previous studies demonstrating that EETs and EET analogs have anti-oxidative effects in several pathological states including diabetic nephropathy [10,13,29]. Again, kidney-targeted EET-F01 had comparable anti-oxidative actions when compared to EET-A. 

EET and EET analogs have been demonstrated to enhance angiogenesis and there is controversy as to whether or not EET analogs could promote tumor growth [14,15,35,36]. Our previous study demonstrated that EET-A does not enhance cancer cell proliferation [30]. In addition, anti-cancer actions for sEH inhibitors that results in increased EET levels have been found in colon, pancreatic, and liver cancer animal models [35,37,38]. Findings in the current study demonstrate that EET-A failed to enhance human breast cancer cell tumor growth in mice. There is also a potential concern with folate conjugation of EET analogs because the folate receptor-α is highly expressed in epithelial, [39,40]. One way to lessen this concern is to develop lysozyme conjugates or dendimers that target the kidney [21,41]. However, our studies have demonstrated that EET analogs do not stimulate tumor growth and that EET analogs did not compromise the anti-cancer effects of cisplatin [26]. Taken together, these past findings and our current findings demonstrate that EET analogs and kidney targeted folate conjugated EET analogs will protect the kidney from cisplatin nephrotoxicity, fail to cause tumor growth, and will not interfere with cisplatin anti-cancer actions.

In summary, the present study provides strong evidence that targeting EET analogs to the kidney is effective in reducing cisplatin nephrotoxicity in rats. Comparison of the kidney-targeted EET analog, EET-F01, to a non-targeted EET analog, EET-A, found that EET-F01 at a significantly lower weighted and molar dose provided a similar reduction in cisplatin nephrotoxicity. We have demonstrated that EET analogs provided kidney protection by the inhibition of inflammatory and oxidative stress pathways that are critically involved in the pathology of cisplatin nephrotoxicity. In conclusion, we have developed a kidney targeted EET analog, EET-F01, that demonstrates excellent potential as a therapeutic for kidney diseases. 

## 4. Materials and Methods

### 4.1. Chemicals

All chemicals and assay kits were purchased from Sigma Aldrich (St. Louis, MO, USA) unless otherwise mentioned. EET analogs were designed and synthesized in the laboratory of John R. Falck, Department of Biochemistry, University of Texas Southwestern Medical Center, Dallas, TX, USA.

### 4.2. EET-F01 Synthesis

**Figure 7:**
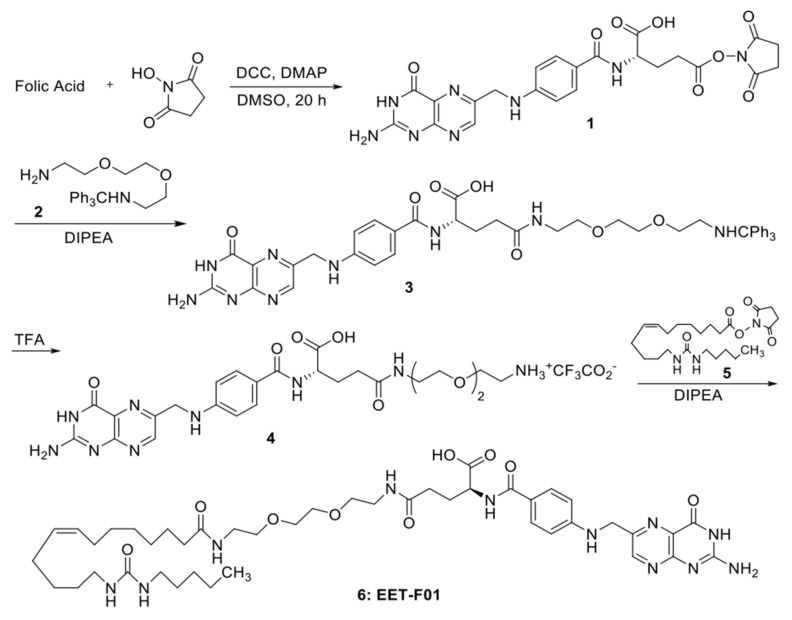
Synthesis of EET-F01.

Diisopropylethylamine (DIPEA, 1.18 mL, 6.78 mmol, 3.00 equiv) was added to a mixture of *N*-hydroxysuccinimide (NHS) folate ester [42] (**1**) (11.44 g, 2.26 mmol) and 2-[2-(2-aminoethoxy)ethoxy]-*N*-tritylethan-1-amine [43] (**2**) (1.06 g, 2.71 mmol, 1.20 equiv) in anhydrous DMSO (20 mL). After 18 h, the DMSO was evaporated (50 °C, 0.1 torr) and the residue was triturated with acetone/ether (30/70, 2 × 50 mL) and then acetone (2 × 50 mL). The residue was dried on high vacuum overnight to give **3** (orange solid, 2.00 g) which was carried on to the next step without further purification. Trifluoroacetic acid (TFA, 5.36 mL) was added dropwise to a suspension of crude **3** (1.09 g, 1.34 mmol) in CH_2_Cl_2_/H_2_O (5/1, 6 mL). After gently shaking for 2 h, the mixture was concentrated in vacuo and the residue was azeotropically dried with dry toluene (10 mL). The residue was triturated with CH_2_Cl_2_ (5 × 5 mL); the yellowish CH_2_Cl_2_ supernatant was removed each time with a pipette and gradually became less yellow with each wash until nearly colorless. The crude 4 (thick red oil) was used in the next step without further purification. DIPEA (1.13 mL, 6.50 mmol, 5.00 equiv) was added dropwise to a suspension of crude **4** (0.891 g, 1.30 mmol) in anhydrous DMSO (6 mL). After 15 min, a solution of EET analog **5 [44]**. (0.569 g, 1.30 mmol) in dry CH_2_Cl_2_ (6 mL) was added. After 24 h, the reaction mixture was diluted with ice-cold 20% acetone/Et_2_O (50 mL). The supernatant was decanted away from the resulting precipitate and the residue was triturated sequentially with additional 20% acetone/Et_2_O (50 mL), 50% acetone/Et_2_O (100 mL), 0.1 N HCl (20 mL), and then acetone (2 × 50 mL). The residue was dried under high vacuum overnight to give EET-F01 (**6**) as an orange solid (0.690 g, 59%) (Figure 7). 

^1^H NMR (500 MHz, DMSO-*d*_6_) δ 8.65 (s, 1H), 7.93–7.80 (m, 1H), 7.73–7.58 (m, 2H), 6.95 (s, 1H), 6.64 (dd, *J* = 8.7, 2.3 Hz, 2H), 5.79–5.69 (m, 2H), 5.38–5.28 (m, 2H), 4.49 (d, *J* = 6.1 Hz, 2H), 3.52–3.45 (m, 4H), 3.22–3.13 (m, 4H), 3.01–2.87 (m, 4H), 2.10–2.09 (m, 2H), 2.05 (t, *J* = 7.5 Hz, 2H), 2.01–1.93 (m, 4H), 1.51–1.40 (m, 2H), 1.40–1.14 (m, 18H), 0.86 (t, *J* = 7.0 Hz, 3H). LCMS (ES-APCI^+^) Calcd. for [C_44_H_67_N_11_O_9_]^+^ 894.1, Found 894.4.

### 4.3. Animals

All animal studies were approved and carried out according to guidelines of the Institutional Animal Care and Use Committee, Medical College of Wisconsin (protocol numbers AUA00001818, 9/5/2019 and AUA00000042, 10/11/2019). Animals were kept in a temperature-controlled environment with a 12-h light/dark cycle and were allowed free access to food and water. An acclimatization period of 6 days was allowed for the rats before experimentation.

### 4.4. Kidney Targeting In Vivo Animal Studies 

Overnight fasted 10 week old male Sprague Dawley rats (Charles River, MA, USA) were anesthetized with sodium pentobarbital (60 mg/kg, i.p.). Following anesthesia, a tracheostomy was performed, and the left jugular vein cannulated to continuously infuse vehicle, EET-A, or EET-F01 at a rate of 6 mL/kg/hr for 6 h. Plasma and kidney samples were collected at the end of the protocol, snap-frozen in liquid nitrogen and stored at −80 °C until analyzed by LC-ESI-MS. EET-A and EET-F01 solutions were prepared in 0.1% DMSO and 1% PEG-400 in saline. 

### 4.5. Mass Spectrometric Analysis 

Plasma and kidney levels of EET-A and EET-F01 were measured by LC–ESI–MS. Samples were prepared from 200 µL of plasma or kidney homogenate from EET-A or EET-F01 treated rats using solid phase extraction with Varian Bond Elut^®^ C18 column (Agilent Technologies, Santa Clara, CA, USA). The extracted samples were stored at −80 °C before analysis. Samples were warmed to room temperature, dried in a stream of nitrogen and the residue reconstituted in 20 µL of acetonitrile of which 12 µL was injected. Components were resolved on a 250 mm × 2.0 mm Kromasil C18-column packed with 5µ diameter particles having 100 Å pores. Gradient elution from 80% A to 10%A was used with eluent flow of 0.2 mL/min. Solvent A was water with 0.01% formic acid and solvent B was acetonitrile with 0.01% formic acid using the following profile: 20%B to 30%B in 10 min, 30%B to 60%B in 17 min, 60%B to 90%B in 28 min, hold at 100%B for 7 min, then 7 min re-equilibration. MS/MS analysis was performed on an Agilent 6460 triple quadrupole mass spectrometer equipped with a Jet Stream™ interface (Agilent Technologies, Santa Clara, CA, USA). Precursor ion, product ion, collision energy and fragmenter voltage were optimized for each compound in negative polarity. Other parameters were as follows: drying gas flow = 10 L/min at 325 °C, nebulizer = 20 psi, sheath gas flow = 11 L/min at 325 °C, capillary = 3.5 kV, and nozzle = 1.0 kV. Results were acquired at unit-mass resolution. 

### 4.6. Cisplatin-Induced Nephrotoxicity Animal Study

Male Wistar-Kyoto (WKY) rats weighing 180–200 g (Charles River, Wilmington, MA, USA) were used. Rats were assigned into groups (*n* = 6 in each group). Treatments were given i.p. in an osmotic pump (2ML, Alzet, Cupertino, CA, USA) starting two days prior to cisplatin injection and included vehicle (20% DMSO in PEG-400 *v*/*v*), EET analog A (EET-A in double distilled water, 10 mg/kg/d), or EET-F01 at 2 mg/kg/d and 20 mg/kg/d doses (prepared in a mixture of 20% DMSO in PEG400), respectively. The control-vehicle rat group received drinking water ad libitum and were administered the same DMSO (300–500 µL i.p.) that was used to prepare cisplatin solutions. Rats in the treatment groups were administered a single dose of cisplatin (7 mg/kg i.p.). One day prior to euthanasia, rat urine was collected over a 24 h period. Five days after cisplatin or vehicle administration, rats were anesthetized for blood and kidney sample collection followed by euthanasia. 

### 4.7. Biochemical Analysis

The levels of blood urea nitrogen (BUN) (BioAssay Systems, Hayward, CA, USA), urinary kidney injury molecule-1 (KIM-1) from R&D Systems (Minneapolis, MN, USA), thiobarbituric acid reactive substances (TBARS) from Cayman Chemical Co. (Ann Arbor, MI, USA), and the activity of urinary N-acetyl-β-(D)-glucosaminidase (NAG) in the urine was measured by a kit (Diazyme Laboratories, Poway, CA, USA). 

### 4.8. Histopathology

A histopathological study was conducted after fixation of the kidneys with 10% buffered formalin. Renal tissues were sectioned and stained with periodic acid-Schiff (PAS) for histological examination. Histological injury was determined in stained tissue sections at magnification of ×200 using image analysis software by NIS Elements AR version 3.0 (Nikon instruments Inc., Melville, NY, USA). To minimize observer bias, histopathological evaluations were conducted by two observers in a masked fashion without knowledge of the treatment group from which the tissues originated. 

### 4.9. Real-Time PCR Analyses

Real-Time PCR (RT-PCR) analyses were carried out to assess renal mRNA expression of the inflammatory gene interleukin-6 (IL-6), tumour necrosis factor α (TNFα) and oxidative stress genes p47phox, gp91phox, Nox4 and p67phox. RNA was extracted from rat kidney tissue using RNeasy Mini Kit (QIAGEN, Germantown, MD, USA). Synthesis of cDNA was carried out from mRNA samples with the iScriptTM Select cDNA Synthesis Kit (Bio-Rad, Hercules, CA, USA). RT-PCR was performed on the cDNA using the iScript 1-step RT-PCR kit with SYBR Green (Bio-Rad, USA) in CXF384 Touch Real-Time PCR Detection System (Bio-Rad, USA). All RT-PCR analyses were performed in triplicate using 384-well plates. Primer sequences are listed in Table 1. The mRNA expression data of target genes were normalized to the expression of a house-keeping gene, expression levels were calculated using the 2 −ΔΔCt method and expressed as fold change vs. normal control group.

### 4.10. Tumor Bearing Studies

We evaluated EET-A on the growth of human breast cancer cell line (MBA-MB-231, basal-like breast cancer cells) in female athymic nude mice (Envigo, Indianapolis, IN, USA). Briefly, 2 × 10^6^ cells in 50 mL of cold phosphate buffered saline was subcutaneously inoculated into 6–8 weeks female athymic mice on both front sides. When tumor growth reached a size of 1000 mm^3^, mice were randomly divided into vehicle or EET-A treated groups with each group consisting of 10 tumors (5 mice) [45]. EET-A (10 mg/kg, p.o.) was given daily for three weeks and tumor volumes were measured every other day. Moreover, tumors at the end of the experiment were weighed and compared between control and the treated group. Mice were euthanized and tumors were collected for histological evaluation. After fixation of the tumors with 10% buffered formalin, tumors were sectioned and stained with periodic Acid-Schiff (PAS) for histological examination. Histological evaluation of the tumors was conducted to determine changes in tumor cellular proliferation. 

### 4.11. Statistical Analysis

Results are reported as mean ± S.E.M. Statistical significance between two measurements was determined by the two-tailed unpaired Student’s *t* test (and among groups it was determined by repeated measure one-way analysis of variance followed by Tukey’s post hoc test) using GraphPad Prism^®^ Version 4.0 software (GraphPad Software Inc., La Jolla, CA, USA). Probability values of *p* < 0.05 were considered significant where the critical value of P was two-sided.

## Figures and Tables

**Table 1:** Primer sequences of the target genes used in RT PCR analysis.

TNFα	F- CGAGTGACAAGCCTGTAGCC R- GAGAACCTGGGAGTAGACAAGG
IL-6	F- GAGACTTCCAGCCAGTTGCC R- TGAAGTCTCCTCTCCGGACTT
p47phox	F- TCGAGAAACGCTTCGTCCC R- GTAGACCACCTTCTCCGACA
gp91phox	F- GGCCCAACTGGGATAACGAG R- TTTTAGCCAAGGCTTCGGGG
Nox4	F- GGCCCAACTGGGATAACGAG R- TTTTAGCCAAGGCTTCGGGG
p67phox	F- GCTTCGGAACATGGTGTCTAAGA R- AGAGTCAGGCAGTAGTTTTTCACTTG
Gpdh	F- AGTGCCAGCCTCGTCTCATA R- GGTAACCAGGCGTCCGATAC

## Data Availability

Data is contained within the article. The data presented in this study are available are available on request from the corresponding author.
